# Fuzzy Logic Type-2 Based Wireless Indoor Localization System for Navigation of Visually Impaired People in Buildings

**DOI:** 10.3390/s19092114

**Published:** 2019-05-07

**Authors:** Basem AL-Madani, Farid Orujov, Rytis Maskeliūnas, Robertas Damaševičius, Algimantas Venčkauskas

**Affiliations:** 1 Computer Engineering Department, College of Computer Science and Engineering, King Fahd University of Petroleum and Minerals, Dhahran 31261, Saudi Arabia; mbasem@kfupm.edu.sa; 2 Faculty of Informatics, Kaunas University of Technology, Kaunas 51386, Lithuania; farid.orujov@ktu.edu (F.O.); rytis.maskeliunas@ktu.lt (R.M.); algimantas.venckauskas@ktu.lt (A.V.)

**Keywords:** indoor positioning, indoor localization sensors, indoor navigation, fuzzy logic, signal fingerprinting, Bluetooth beacons, assisted living

## Abstract

The ability to precisely locate and navigate a partially impaired or a blind person within a building is increasingly important for a wide variety of public safety and localization services. In this paper, we explore indoor localization algorithms using Bluetooth Low Energy (BLE) beacons. We propose using the BLE beacon’s received signal strength indication (RSSI) and the geometric distance from the current beacon to the fingerprint point in the framework of fuzzy logic for calculating the Euclidean distance for the subsequent determination of location. According to our results, the fingerprinting algorithm with fuzzy logic type-2 (hesitant fuzzy sets) is fit for use as an indoor localization method with BLE beacons. The average error of localization is only 0.43 m, and the algorithm obtains a navigation precision of 98.2 ± 1%. This precision confirms that the algorithms provide great aid to a visually impaired person in unknown spaces, especially those designed without physical tactile guides, as confirmed by low Fréchet and Hausdorff distance values and high navigation efficiency index (NEI) scores.

## 1. Introduction

Currently, there is increasing interest in obtaining information about the location of an object, especially in the area of navigation solutions for visually deficient or impaired people [1]. The range of services will expand significantly if a user’s location information can be provided. The location-based services refer to applications that depend on the user’s location to provide services in various categories, including navigation and tracking, leading to the enormous social and economic potential of indoor positioning services (IPS). Adaptive navigation technologies can enhance indoor way finding by visually impaired people [2]. Unfortunately, the Global Positioning System (GPS) technology does not specify whether a location is close to walls, buildings, trees, buildings, and subways, as the power of the GPS satellite signal is weak, making it unusable for indoor GPS localization. It is common to use WiFi hotspots for detecting location in the indoor environment (such as office buildings, industrial facilities, or smart homes). However, since walls are obstacles that affect the signal WiFi access points, that data mechanism is not effective. In this case, the quantity and location of WiFi access points are very important when using wireless technology; moreover, such a solution is costly. WiFi-based fingerprinting can achieve good accuracy (up to 1.21 m with an accuracy of 98%), but it is slower (takes 5.43 s to estimate, while proximity-based solutions are cheaper, do not require calibration, and offer good accuracy [3]. While there is a possibility of using a sort of guide based on an image recognition [4], these systems need to locate some sort of markers or objects and target a camera at them, which is not feasible for a visually impaired person [5]. This problem could be partially solved by machine vision [6]. Most non-wireless systems refer to some sort of proprietary hardware, e.g., microelectromechanical systems (MEMS) [7]; radio-frequency identification (RFID) systems [8] are very rare in artificial laboratory environments and in reality.

The main task of indoor navigation is locating people or objects in indoor environments such as public buildings. The main challenges for indoor navigation are building and maintaining accurate maps; the availability of technology (sensors, devices) for localization, signal interference (reflection, attenuation, multipath, and blockage), and the calibration of equipment to collect enough measurement samples for actual use; and use in uncontrolled environments where the users have no control over the placing of the equipment that is necessary to support indoor positioning.

Current approaches to indoor localization can be categorized as infrastructure-based and infrastructure-less. Infrastructure-based approaches utilize and heavily depend upon the existing infrastructure such as WiFi or Global System for Mobile Communications (GSM). For example, Du et al. [9] used crowdsourced WiFi signal data and built-in smartphone sensors to achieve high-positioning accuracy and low power consumption, which outperformed the GPS-based method. Huh and Seo [10] used Bluetooth 4.0, received signal strength indication (RSSI), Bluetooth Low Energy (BLE), and trilateration to determine the position of the user in an indoor space. Kawai et al. [11] used BLE RSSI data processed by multilayer perceptron (MLP) with an extended Kalman filter, reaching an error of 2.21 m. Lee et al. [12] used Beacon to transmit a radio frequency (RF) signal and an audio signal of specific frequency. The smartphone calculates distance values using the Time Difference of Arrival (TDoA) method and uses them for trilateration. Naz et al. [13] used Visible Light Communication (VLC), which includes both frequency and the variable phase of the transmitted signal, estimating the position of an object with a localization error when the signal passes through an optical channel, and achieving positioning accuracy within 1–2 cm. Uradzinski et al. [14] used Zigbee wireless technology and employed data filtering, weighted k-nearest neighbors (KNN), and the Bayesian algorithm to calculate the pedestrian’s location, obtaining the average error less than or equal to 0.81 m. 

Navigation cues can be provided using statistical methods applied on static floor fields in open space rooms [15]. A virtual graph based on luminaire analysis was suggested as another possible approach in [16]. Segura et al. [17] used the ultra wideband (UWB) system for indoor navigation and achieved a positioning error of 20 cm (the anchor method by Großwindhager et al. [18] also showed an error of 20 cm); however, this method is more difficult to apply (comparing to BLE and WiFi-based methods) due to the requirement of having specific equipment, which may not be available to regular users. Zhang et al. [19] proposed the use of a combination of GPS, UWB, and MARG (magnetic, angular rate, and gravity), achieving a positioning error is 3.2 m, which might not be acceptable for a blind person. Zhou et al. [20] proposed a combined method based on images from a smartphone camera capturing the surrounding scene and pedestrian dead reckoning (PDR) to determine the pedestrian’s trajectory with an accuracy of about 0.56 m. Using embedded inertial sensors [21] and PDR [22] by updating the current position through measuring the length and title of each step, enabled reaching an error of 1.96 m. A similar multisensory approach improved this up to 1.46 m [23]. A combination with stereo cameras has improved the accuracy up to 0.677 m [24]. A combination with Bluetooth beacons can provide an average error rate around 2.53% [25]. 

The placement and density of signal transmitters such as BLE beacons may have a crucial impact on the accuracy of indoor localization. For example, Rezazadeh et al. [26] proposed an improved beacon placement strategy that enables 21.7% higher precision than using normal iBeacon placement. 

Using the available map of a building or facility provides additional useful information for accurate indoor localization. For example, Wang et al. [27] proposed a scheme for determining the location in the room by combining the floor map, WiFi data, and smartphone sensors with an average error of 3.135 m for KNN, and 4.99 m for PDR. A similar method in combination with the fingerprint algorithm allowed a reduction of around 37% [28]. Xu et al. [29] proposed using a grid-based indoor model to create a floor plan to track indoor location with an average accuracy of 92%. The RSSI combination method applied on BLE beacons with cartographic information without using the value of the beacon distance showed an error rate of 1.6 m [30].

Infrastructure-less approaches require no support from the existing infrastructures or networks such as internet access points (APs). For example, Jeong et al. [31] used only a smartphone as a mobile beacon that is capable of tracking its own position by using its motion sensor data. The smartphone broadcasts short-distance beacon messages and collects response messages from neighboring Internet of Things (IoT) devices along with the message’s signal strength and its position, thus obtaining less than a 20-cm position error in a real-world setting. Link et al. [32] adopted sequence alignment algorithms from the field of bioinformatics for the accurate localization of a subject using only accelerometer and compass sensor data from a smartphone.

The fingerprinting technique, which has been used for both infrastructure-based and infrastructure-less approaches, involves the use of measurements (aka fingerprints) of some physical quantity such as received signal strength indication (RSSI). Tomazic et al. [33] improved the fingerprinting method with the interval fuzzy model to calculate the confidence interval for the k-nearest neighbors (kNN) search in the database of fingerprints, thus achieving an improvement of localization results by 40%. Dari et al. [34] used the received signal strength (RSS) received from the access point (AP), and applied the location fingerprint technique using the features of RSS’s fingerprint, while the position was determined by the k-nearest neighbor (KNN) method. Cha and Xiaoran [35] used Naïve Bayes and WiFi fingerprinting for indoor localization. The router is used as the generator of the WiFi signal, the Naive Bayes models train the data, and the server calculates the position, reaching an accuracy of more than 80%. Raspopoulos [36] used device-independent radio maps generated by deterministic channel modeling through three-dimensional (3D) ray tracing (RT) for WiFi RSSI-based fingerprinting. Song et al. [37] proposed a channel state information (CSI) amplitude fingerprinting-based localization algorithm and multidimensional scaling (MDS) to calculate the Euclidean distance and time-reversal resonating strength (TRRS) between target and reference points. Finally, the KNN algorithm was used for location estimation. The final estimated position is obtained by the results of MDS and KNN, which reduces the positioning error. Subedi and Pyun [38] improved traditional fingerprinting localization by combining it with weighted centroid localization (WCL), which allowed reducing the number of required fingerprint reference points by more than 40% while maintaining a similar localization error. 

Surrounding walls, equipment, and obstacles, including human bodies, can attenuate and distort wireless positioning signals. The problem of obstacles was addressed in Wang et al. [39]. The authors estimated the distance using the time-of-arrival (TOA) measurement model and applied residual analysis to identify the non-line-of-sight (NLOS) error. Finally, the particle swarm optimization with a constriction factor (PSO-C) was used to compute the position. A method to detect and prevent collisions with obstacles, based on the Kalman filter algorithm with time stamps (TSM-KF) using an RGB-Depth (RGB-D) camera was suggested by [40]. Deng et al. [41] focused on the problem of the body-shadowing impairment of RSS-based positioning, and derived a mathematical relation between the body-shadowing effect and positioning error. 

The use of multiple data sources may require employing data fusion to produce more consistent and accurate results. Al-Qudsi et al. [42] performed a fusion of data from a multi-band frequency modulated continuous wave (FMCW) radar system using a particle filter-based tracking method, and achieved a positioning error of less than 17 cm and 31 cm for outdoor and indoor conditions, respectively, outperforming the commercial systems. Seco and Jimenez [43] combined the RSS of RF signals emitted from known location beacons together with combined with pedestrian dead reckoning (PDR) estimates of user walking. A centralized cooperative particle filter (PF) was applied to improve the localization result, reaching a location error of 1.6 m. Widyawan et al. [44] employed the backtracking particle filter (BPF) to improve indoor localization performance, achieving up to 25% improvement.

An improvement of the results of localization can include a variety of the techniques. The examples include error compensation for the body-shadowing effect [45], continuous feature scaling and outlier deletion [46], collaborative localization using information from multiple nodes [47], error correction using collision avoidance velocity and map-aided inertial dead reckoning (DR) [48], probabilistic fingerprint (P-FP) using the probability density functions of the received signal strength algorithm (RSSA) [49], the use of optimization algorithms to decrease the localization error considering different RSS thresholds for hybrid indoor positioning [50], and particle swarm optimization (PSO) for fitting the signal attenuation curve, thus allowing the developed parametric model to locate the user’s position with the standard deviation of positioning of 1.15 m [51]. 

For a survey of the remaining works in the area, the readers can consult the recent surveys in [52,53,54].

In our previous article [55], we presented the results of a series of experiments using signal strength received from the Bluetooth Low Energy (BLE) beacons and applied various positioning algorithms such as proximity, centroid localization, weighed-centroid localization, fingerprinting, etc. to determine the effect on positioning error. Then, we proposed a fuzzy logic [56]-based scheme to select the most fitting positioning algorithm depending upon the strength of the signal, the number of beacons available, and the size of the room. 

In this paper, we combined fuzzy logic type-2 for indoor positioning in the real-world environment, allowing a flexibility of complex environments (glass/metal corridors) such as our test building. We have adopted multi-fuzzy sets-based membership location distance methods and compared the results of using fuzzy logic type-1 and fuzzy logic type-2 with those obtained without using fuzzy logic.

## 2. Materials and Methods

### 2.1. Model of Indoor Localization

**Figure 1:**
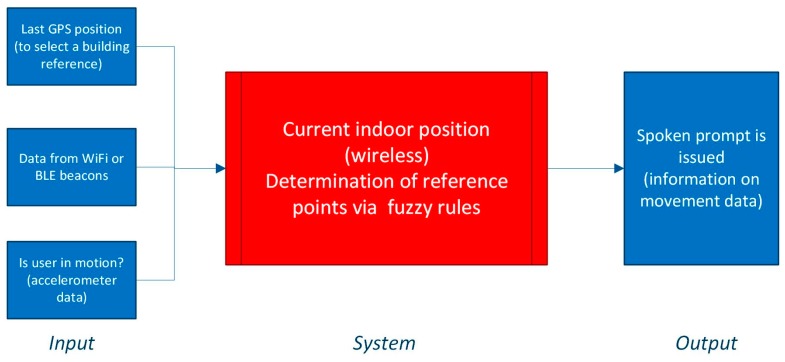
Model of indoor navigation system for blind people.

Our indoor localization model is based around a smartphone for full and complete processing. The system selects the reference data for a building (site location) based on a valid GPS coordinate, as data from a mobile network transferred using General Packet Radio Services (GPRS) can quite accurately identify the exact building. Once started, the algorithm constantly reads data from environmental sensors (WiFi and BLE beacons). The model of the environment includes several beacons and a smartphone as the receiver. Without loss of generality, the space is regarded as a flat environment in which there are interferences from walls, floors, diverse signals, etc. Three versions of the fingerprinting algorithm will be considered: (1) without using fuzzy logic, (2) using fuzzy logic type-1; and (3) using fuzzy KNN and fuzzy logic type-2. Once the location is identified or a change is detected (motion), a spoken prompt is given to a user telling his location. All of the system processing was done on an Android smartphone. The overall model is illustrated in Figure 1.

### 2.2. Fingerprinting Localization

**Figure 2:**
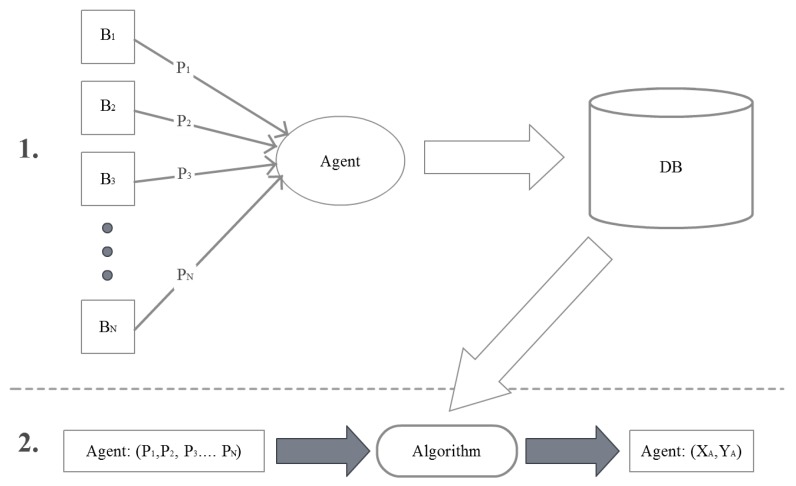
The scheme of the fingerprinting localization algorithm.

The algorithm [57] is based on the spatial signature signal differentiation. The location of the receiver is determined by comparing the currently measured signature signal power with signatures of location points pre-formed and stored as a database. Figure 2 shows the two phases of the algorithm:

- The stage configuration environment. At this stage, the power signals of all the known active beacons are measured in pre-planned locations. The information collected is stored in a database with reference to the local coordinate space (assigned to specific rooms) or global coordinate space (assigned to a building).
- Step positioning. At this stage, the signal power measurements made over the receiver are compared with the information stored in the database by means of an algorithm. The k-nearest neighbors algorithm is used [58].

When creating a fingerprint point, a scan and a collection of signals from beacons were performed within 10 s. The average RSSI signal for each beacon was calculated and saved to the set.

The average RSSI value for each beacon was calculated using the formula:

(1) $$RSSI_{i}=\sum_{j=0}^{N}\frac{RSSI_{ij}}{N}$$

where $RSSI_{i}$ is the signal strength of the *i*-th beacon, $RSSI_{ij}$ is the instantaneous power of the signal, and N is the number of measurements within 10 s of measurement.

#### 2.2.1. K-Nearest Neighbors

Formula (1) is used to find the Euclidean distances between the stored data and real-time data:

(2) $$D_{i}=\sqrt{\sum_{j=1}^{k}{\left(P_{ij}-P_{i}{}^{\prime }\right)}^{2}},$$

where *i* is *i*-th pre-planned location point; *P_ij_* is the RSS from the *i*-th beacon in the *j*-th pre-planned location point, which is stored in a database; and *P*’*_i_* the is real-time incoming RSS from the *i*-th beacon.

In the next step, one pre-planned location point is selected with the smallest Euclidean distance. The value of the coordinates of the pre-planned location points are assigned to the coordinates of the receiver. The k-nearest neighbors (KNN) algorithm is used for choosing a multitude of pre-planned location points. The nearest neighbor (NN) algorithm is a special case of KNN, when *k* = 1. The advantage of using multiple points is to improve the positioning accuracy. There is a possibility of using additional algorithms to approximate the location. The authors of [59] suggest using weighted centroid localization. They used *k* = 4. The coordinates of the receiver are found by:

(3) $$X_{0}=\sum_{i=1}^{k}w_{i}X_{i},Y_{0}=\sum_{i=1}^{k}w_{i}Y_{i},$$

where $X_{0}$, $Y_{0}$ are the Cartesian coordinates of the receiver; $X_{i}$, $Y_{i}$ are the Cartesian coordinates of the *i*-th beacon; k is the number of pre-planned location points; and $w_{i}$ is the weight.

#### 2.2.2. Fuzzy Logic Type-1

Fuzzy logic type-1 was implemented using multi-fuzzy sets [60] as an extension of traditional fuzzy set theory in terms of multi-membership location distance functions. Let X be a nonempty set, J be an indexing set, and $\left\{L_{j}:j\in J\right\}$ be a family of partially ordered sets. A multi-fuzzy set A in X is $A=\left\{h_{x},\left(\mu_{j}\left(x\right)\right)\;j\in J_{i}:x\in X,\mu_{j}\in L_{j}X,j\in J\right\}.$, where $h_{x}$ is a nonempty closed interval. The indexing set J may be uncountable. The function $\mu A=\left(\mu_{j}\right)\;j\in J$, J={1,2,…,n} is the membership function of A, and $Q_{j}\in J_{L_{j}}$ is the value domain. The distance membership function $\mu A=h\mu_{1},\mu_{2},\ldots$ is a sequence having precisely n− terms and $L_{j}=\left[0,\;1\right],$ then $M^{n}F_{S}\left(X\right)$ denotes the set of all the multi-fuzzy sets in X.

**Figure 3:**
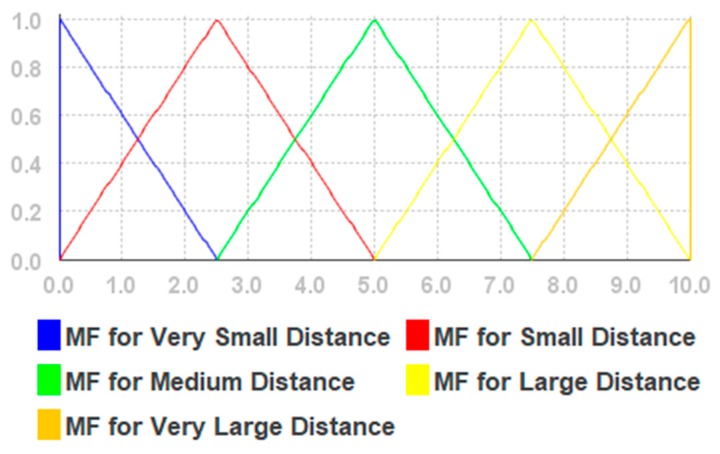
The membership functions for Euclidean distances.

**Figure 4:**
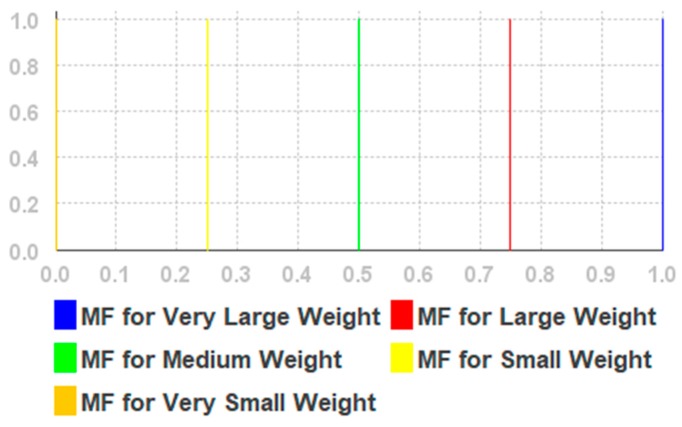
The membership functions for weight.

Based on our previous research results presented in [55], extensive pre-experiments, and an evaluation of the fuzzy membership functions presented in [61], we use triangular membership functions for each linguistic variable. The values and type of the function were chosen heuristically. The simplified fuzzy rules are given in Table A1 in Appendix A, (for Euclidean distance), Table A2 (for Weights) in Appendix C, and illustrated in Figure 3 and Figure 4. The fuzzifier is a triangular membership function. The inference engine is the Mamdani max–min. The defuzzifier is the height method.

The coordinates of the receiver are calculated by the following formulas:

(4) $$X_{0}=\frac{\sum_{i=1}^{k}w_{i}X_{i}}{\sum_{i=1}^{k}w_{i}},Y_{0}=\frac{\sum_{i=1}^{k}w_{i}Y_{i}}{\sum_{i=1}^{k}w_{i}}$$

However, experimentally, we found that when calculating Euclidean distance (RSSI values) at points that are in the diametrically opposite direction, they have the same value. Thus, an error in accuracy increases in the stage of coordinates determination using KNN for *k* > 1.

Here, we propose a new fuzzy mechanism for calculating the Euclidean distance for the subsequent location determination (Equation (4)). It is similar to the Mahalanobis distance; however, instead of the weights purely defined by the data from the covariance matrix, more flexibility is introduced, which allows achieving better results in the end. The formula for calculating the distance is:

(5) $$D_{i}=\sqrt{\sum_{j=1}^{k}{\left(\left(P_{ij}-{P^{\prime }}_{i}\right)\cdot w_{ij}\right)}^{2}}$$

where *i* is the *i*-th pre-planned location point; $P_{ij}$ is the RSS from the *i*-th beacon in the *j*-th pre-planned location point, which is stored in a database; ${P^{\prime }}_{i}$ is the real-time incoming RSS from the *i*-th beacon; and $w_{ij}$ is the additional weight obtained by the fuzzy inference system (FIS).

We propose using the BLE beacon signal strength and the geometric distance from the current beacon to the fingerprint point. The strength of the signal depends upon many factors, such as competing signals, and the material and composition of physical barriers (such as walls, etc.) [62]; therefore, it is difficult to model it reliably. The crisp output variable with the weight of Euclidean distances is denoted by w. Distance membership functions have values from 0 to 10. The values of RSSI membership functions have values from −90 to −20. Weight membership functions have values from 0 to 1. 

**Figure 5:**
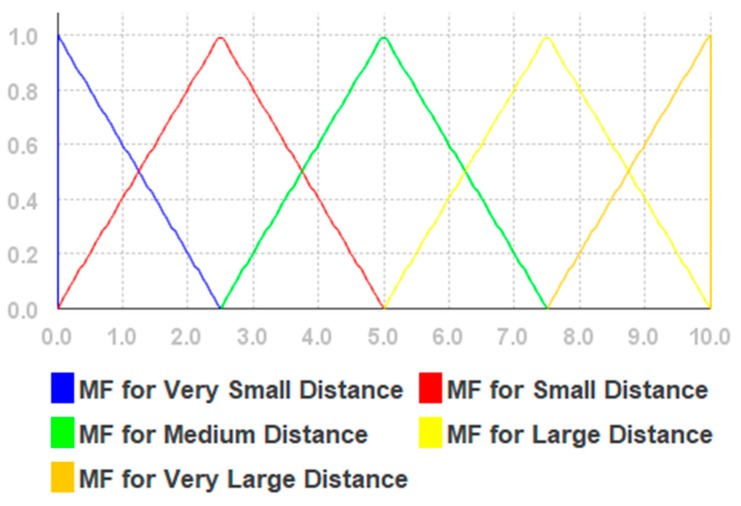
The membership functions for geometrical distance.

**Figure 6:**
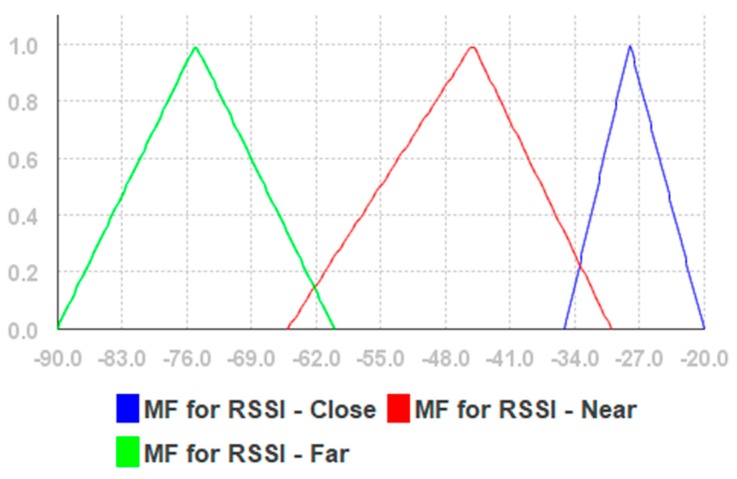
The membership functions for received signal strength indication (RSSI).

**Figure 7:**
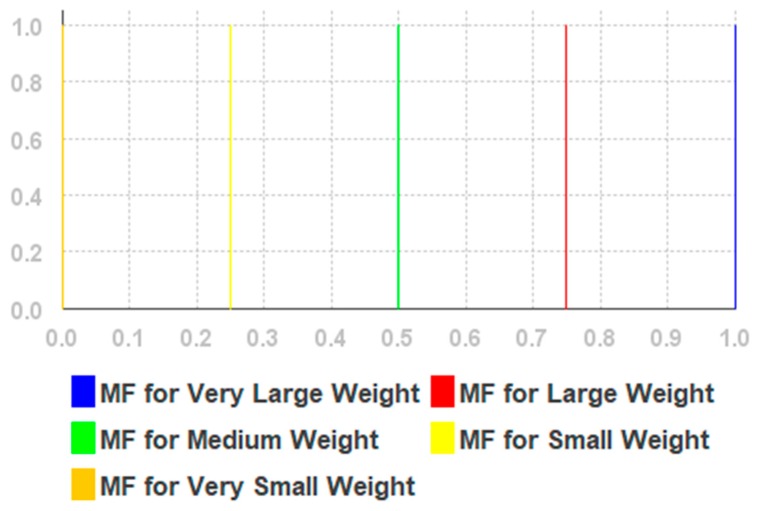
The membership functions for weight.

The fuzzy rules are given in Appendix B and the linguistic variables are given in Table A2, Table A3 and Table A4 of Appendix C. We used triangular membership functions for each linguistic variable, while variable values were selected based on our previous research reported in [55]. The membership functions and fuzzy singleton are shown in Figure 5, Figure 6 and Figure 7, respectively. 

Since the values of the Euclidean distance will be floated in this implementation, we proposed using fuzzy logic type-2 for flexible positioning system adjustment (see the next section).

#### 2.2.3. Fuzzy Logic Type-2

Fuzzy logic type-2 rules were built by introducing a hesitant fuzzy set approach [63]. The fuzzifier was based on upper and lower type-1 triangular membership functions. The inference engine was the Mamdani max–min. The defuzzifier is a centroid method. In the implementation, we can define a hesitant fuzzy set as a function that maps the signal source elements in the area to a set of membership values. If X is a reference set, then a function h when applied to a hesitant fuzzy set X returns a subset of [0, 1]. Let $M=\left\{\mu_{1},\;\mu_{2},\ldots ,\;\mu_{N}\right\}$ be a set of N membership functions. Then, we can define the hesitant fuzzy set associated with M, as:

(6) $$h_{M}\left(x\right)=\underset{\mu \in M}{\cup }\left\{\mu \left(x\right)\right\}$$

In the case of the output of our system, say that R is a set defined in Appendix A. The outcome of the fuzzy rule-based system with the input variable $x_{0}$ is:

(7) $$\hat{y}\left(x_{0}\right)=\underset{\mu }{\cup }A_{i}\left(x_{0}\right)\wedge B_{i}.$$

where fuzzy sets $A_{i}$ and $B_{i}$ are the memberships in the distance domains, and $\hat{y}\left(x_{0}\right)$ is the hesitant fuzzy set associated with ${\left\{\mu A_{i}\left(x_{0}\right)\wedge B_{i}\right\}}_{i}.$

**Figure 8:**
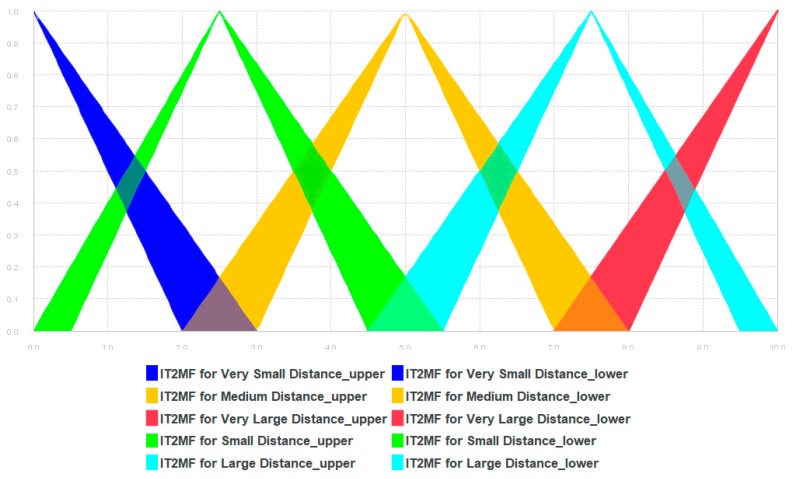
The membership functions for Euclidean distances.

The end rules are given in Table A6 of Appendix C and illustrated in Figure 8.

### 2.3. Evaluation

To evaluate the error of the measured navigation path with respect to the true navigation path, we use Hausdorff and Fréchet distance metrics normalized by the total length of the true path and multiplied by 100% (relative Hausdorff and relative Fréchet). The Fréchet distance measures the shape difference and distance between two trajectories [64], while the Hausdorff distance is the largest distance of a set representing curve points to the closest point in another set of points [65]. The Hausdorff and Fréchet distances are better suited to measure the geometric similarity between paths than the Euclidean distance. 

To evaluate the positioning error, we use the cumulative distribution function (CDF) of the positioning errors. The CDF value has been used previously to evaluate the distribution of the positioning errors [66].

We have evaluated the navigated paths using a navigation efficiency index (NEI) based on [67]. The NEI is the ratio between the actual path traveled and the target path, which is considered to be optimal. The average NEI is calculated on sub-paths, i.e., a part of the path taken by the subject while walking from the beginning to the end of the path as follows:

(8) $$\mathrm{NEI}=\frac{1}{N}\sum_{i=1}^{N}\frac{L_{A}\left(S_{i}\right)}{L_{O}\left(S_{i}\right)}$$

where *N* is the number of sub-paths, $S_{i}$ is a sub-path, $L_{A}$ is the actual length traveled, and $L_{O}$ is the optimal length of $S_{i}$.

Following [68], we also calculate the relationship between localization error and beacon density. The relationship is calculated by removing the beacons one by one and recalculating the average localization error. 

## 3. Experiments and Results

### 3.1. Experiment Setting

The fingerprinting localization algorithm was tested in an environment where a room uses six beacons. The BLE beacon has been configured to have a transmission power (Tx) of 4 dBm, and the advertising interval (i.e., an interval between subsequent transmissions of the advertising packet by the beacon) of 200 ms. The beacons are mounted on walls at the same height. We have removed all the objects that could reflect or absorb signals from the room. The room is a specific environment of 4.64 m × 4.64 m, which has been used to test the algorithms. Beacons were installed within the three-meter range, as it was found to produce the best results in our previous experiments [55], i.e., the distance between the beacons in the room did not exceed three meters. Installation sites were selected in such a way that they were not covered by other objects. Changes in the signal from overlapping beacons were also considered.

### 3.2. Results of Fingerprinting Localization

**Figure 9:**
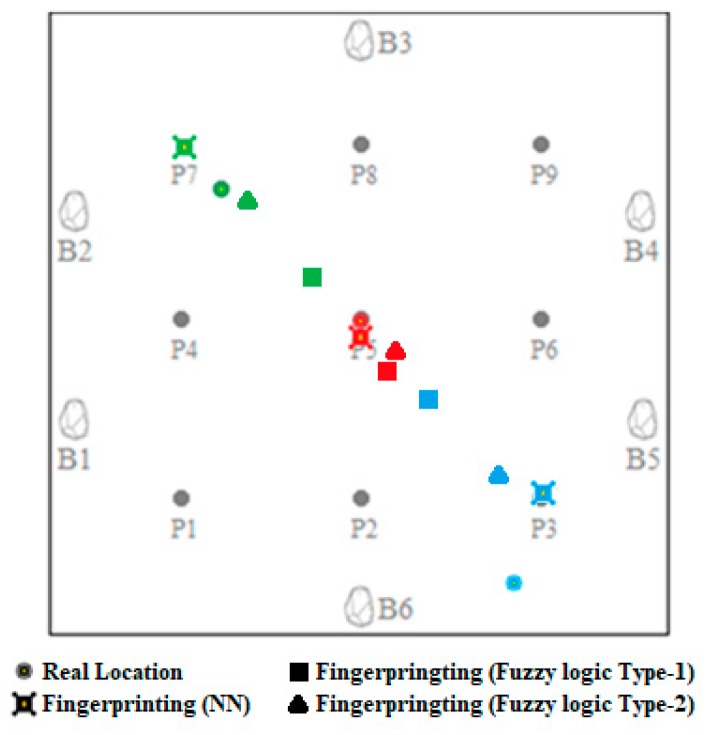
The visual result of the fingerprinting localization algorithms.

During the experiments, the points were randomly chosen, which ought to determine the location of a blind person striving across the environment. Figure 9 illustrates the visual result of the fingerprinting localization algorithm performed in a real-world environment.

The results of the fingerprinting localization algorithm using KNN, where *k* = 1, requires preliminary measurements, which are as follows: the average deviation from the actual position is equal to 0.31 m for the red point, 0.48 m for the green point, and 0.75 m for the blue point. The fingerprinting localization algorithm using fuzzy logic type-1, where *k* > 4, showed that the average deviation from the actual position is equal to 0.38 m for the red point, 0.78 m for the green point, and 0.95 m for the blue point. The fingerprinting localization algorithm using fuzzy logic type-2, where *k* > 4, shows that the average deviation from the actual position is equal to 0.34 m for the red point, 0.23 m for the green point, and 0.77 m for the blue point. 

**Figure 10:**
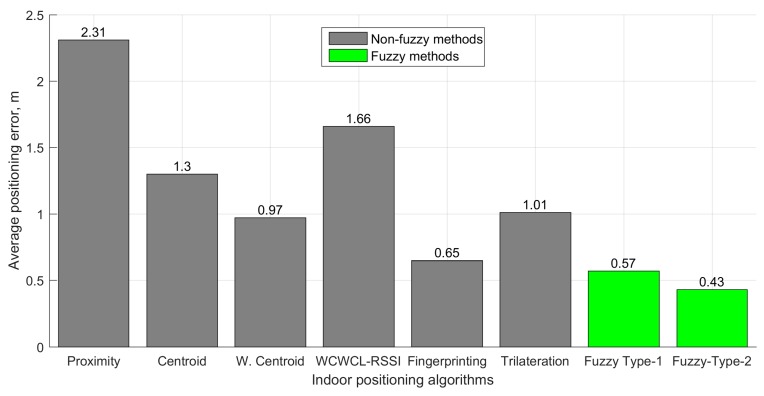
The comparison of the error of indoor positioning algorithms in the rooms.

As we can see from Figure 10, the fuzzy logic type-2 based method for fingerprinting localization achieved an average error of 0.43 m, whereas in case of the non-fuzzy fingerprinting localization algorithm, the average error was 0.65 m, while other non-fuzzy methods (proximity [69], centroid [70], weighted centroid [71], (weight-compensated weighted centroid localization based on RSSI (WCWCL-RSSI) [72], and trilateration [73]) performed even worse (the results of non-fuzzy methods are reproduced from [55]). 

### 3.3. Real-World Experiment

**Figure 11:**
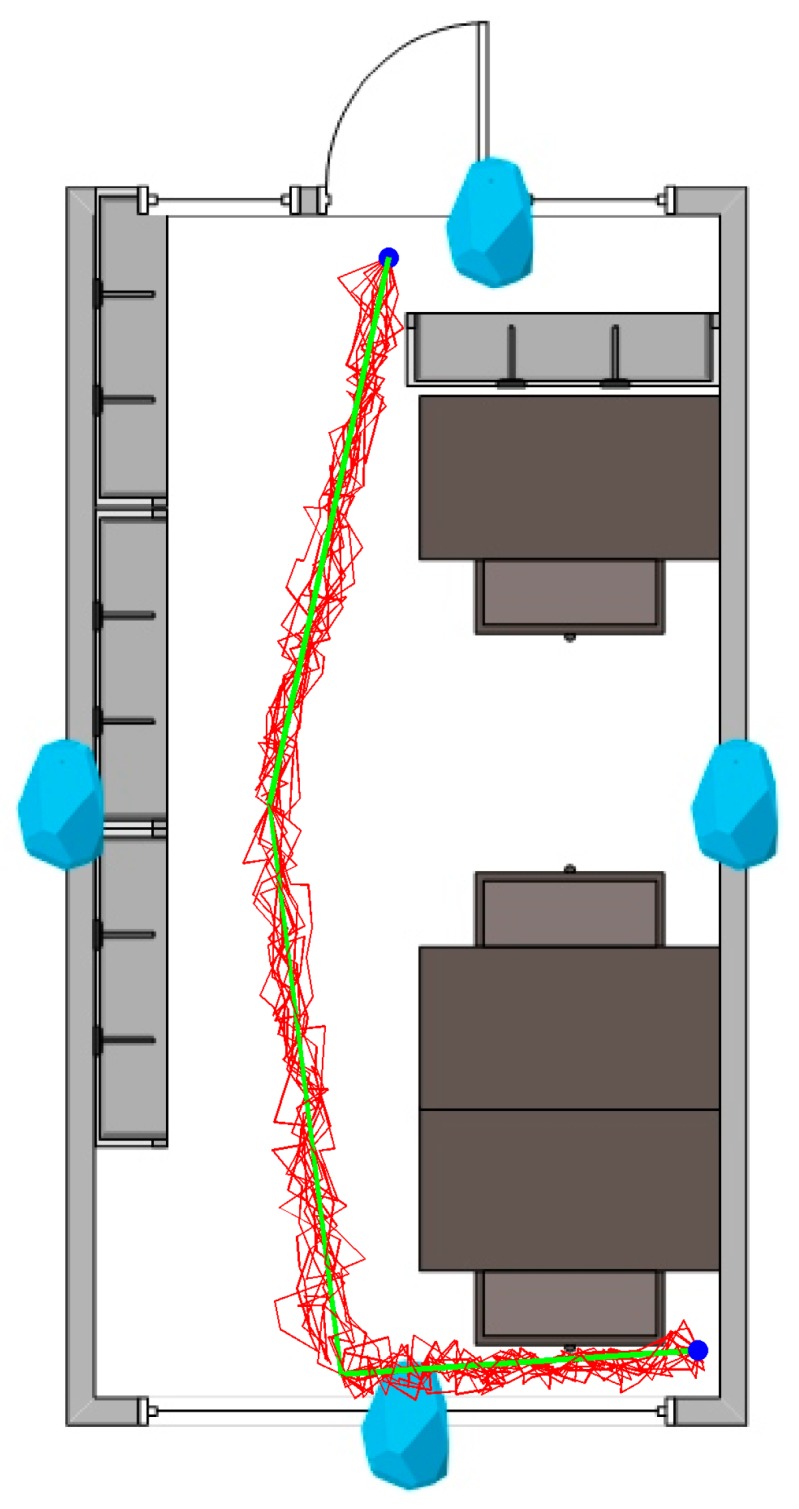
Plan of a representative room with Bluetooth Low Energy (BLE) beacons installed. The person is following the green (true) path, from the top to the bottom of the map. Registered paths are shown in red.

The system was tested with a simple scenario of navigating a corridor and the working environments of our location in the Santaka Valley building, Kaunas, Lithuania. The rooms in the upper row were 3.5 × 4.5 m. The rooms in the bottom row were 3.5 × 6.5 m. Two walls (entry) and the window were made by aluminum-framed glass, while the other two were composed of sound insulated plasterboard sandwich (with a metal construction in between). All the rooms had operational computing and other digital equipment, as the research aim was to investigate the usability of our method in realistic conditions. For indoor positioning, four Bluetooth Estimote beacons were used (we followed the one beacon per wall rule). Each one was mounted at a height of 1.5 m, opposite of each other (see Figure 11). The developed system indicated a distance to the wall and an opening space, as well as the identification number (ID) of the current room or area, all assisting a person with identifying his or her location. The ground truth was established using an overhead mounted camera, while the true path was registered by following the center of the subject’s head. 

For the task of path following (see Figure 11), we achieved a navigation precision of 98.2 ± 1%.

**Figure 12:**
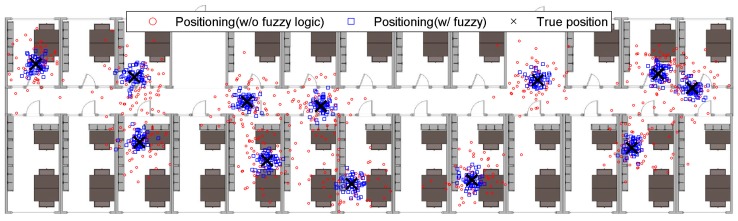
Plan of rooms in the building with positions of subjects established without using fuzzy rules and with the use of fuzzy rules vs. true position.

Thirty-two subjects (17 male, 15 female, aged 19–45 years) were enrolled for the experiments. All the subjects were healthy people without known motoric disorders. The experiment consisted of two sessions. In first session, the subjects were standing in one location without movement for 5 min in the predefined positions (eight—inside rooms, three—at the corridor between the rooms), and their location was established and compared with and without fuzzy logic rules (Figure 12). 

**Figure 13:**
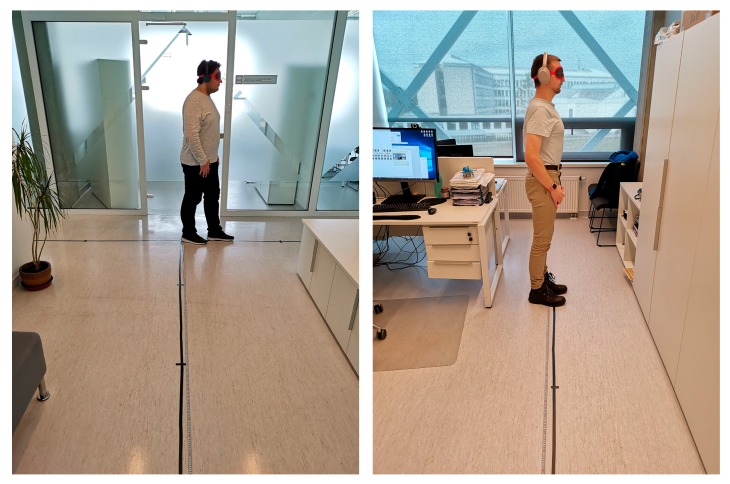
Photos of corridor and room in the building taken during the experiments: note the path marked by tape and reference points (14 on the left, 13 on the right).

In the second session, the point of origin was marked in the room at the end of the corridor, and 20 two-dimensional (2D) points of measure (x and y) among the path were marked on the floor for reference (similarly as in Microsoft Indoor Localization Competition [74]). The ground truth measurements of the evaluation points were set using a measuring tape and a 90° cross-angled laser level. The accuracy of each reference point was no more than a millimeter per 5 m. The subjects were walking blindfolded while only listening to navigational instructions (go, stop, turn left/right) to always follow the same predefined path of the reference points (see Figure 13). 

**Figure 14:**
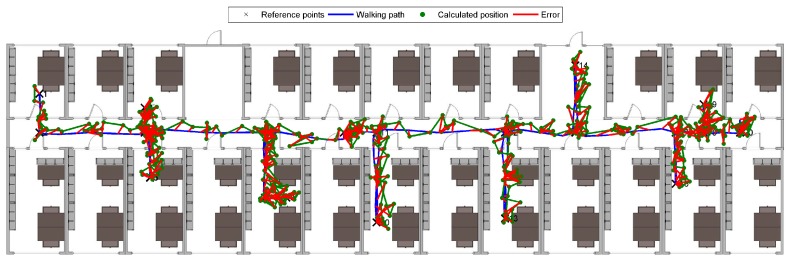
Positions and errors of subjects calculated while walking blindfolded and following audible instructions.

The established positions on a planned walking path versus the “true” subject positions on the walking path and corresponding errors (defined as the Euclidean distance between the true and reported coordinates) are represented in Figure 14.

**Figure 15:**
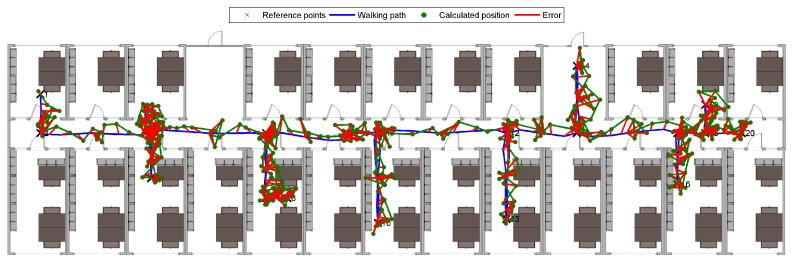
Positions and errors of subjects calculated while visually following the path.

We have remeasured the same experiment walking non-blindfolded, but instructing participants to visually follow the path on the floor (placing feet directly on the contrasting tape). The established positions on a planned walking path versus the “true” subject positions on the walking path and corresponding errors are represented in Figure 15. No statistically significant difference (using the ANOVA test) between the results of the two experiments was found.

### 3.4. Evaluation

**Figure 16:**
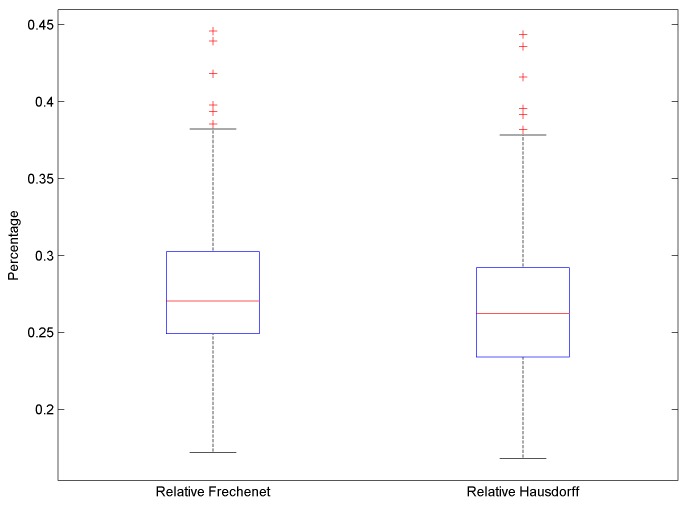
Path error between the “true” path and registered paths.

The measurement results are presented in Figure 16. The average error distance metrics do not exceed 0.27%, while the maximal error does not exceed 0.45%.

**Figure 17:**
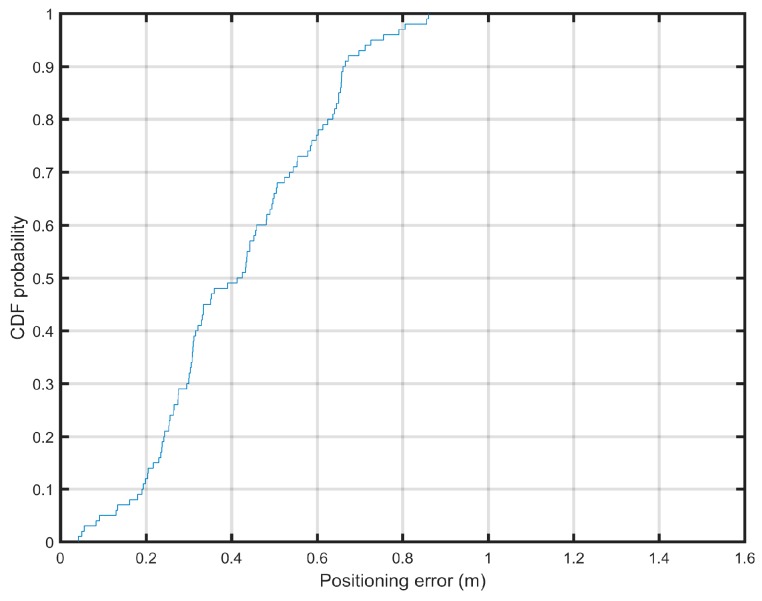
Cumulative distribution function of the positioning error.

Figure 17 shows the CDF of the positioning errors. Our method enables achieving less than 0.63 m of error in 90% of measurements.

**Figure 18:**
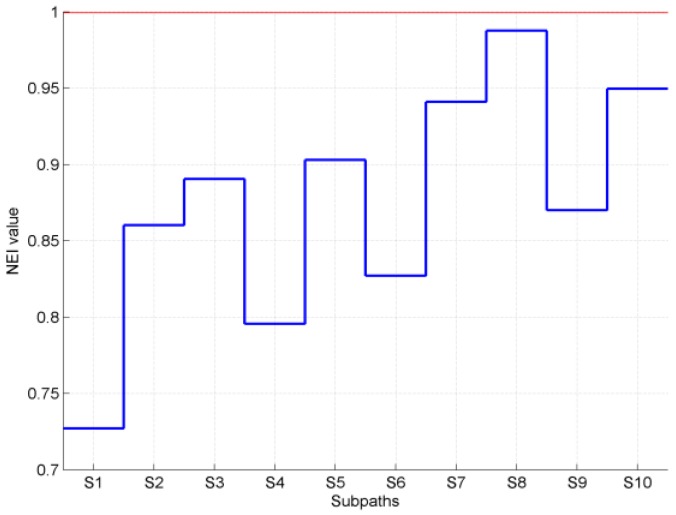
Mean navigation efficiency index (NEI) versus sub-paths (S).

**Figure 19:**
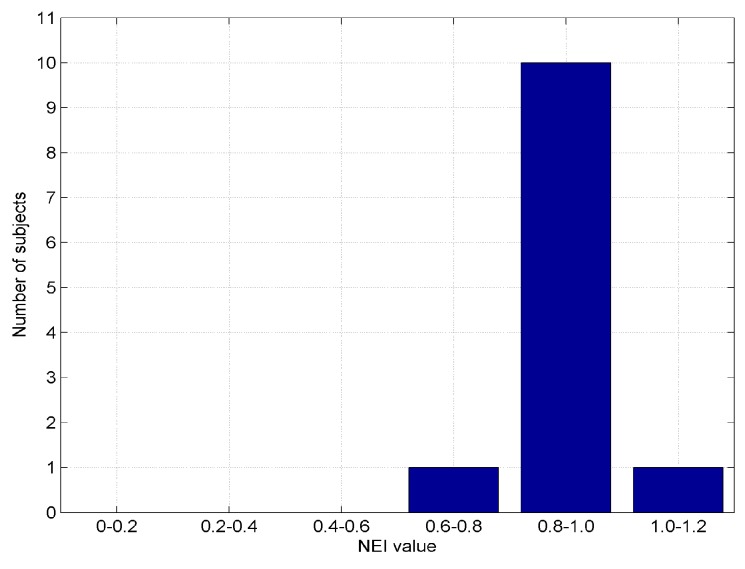
Distribution (histogram) of NEI values.

We have evaluated the navigated paths using a navigation efficiency index (NEI). In this case, we had the main path divided into 10 sub-paths. The results are given in Figure 18 and Figure 19. Note that for some sub-paths, the NEI may be higher than one, because the subjects are allowed to take shortcuts instead following the predefined path. The measured NEI score shows that the usability of this system is indeed acceptable in the tested indoor navigation scenarios.

**Figure 20:**
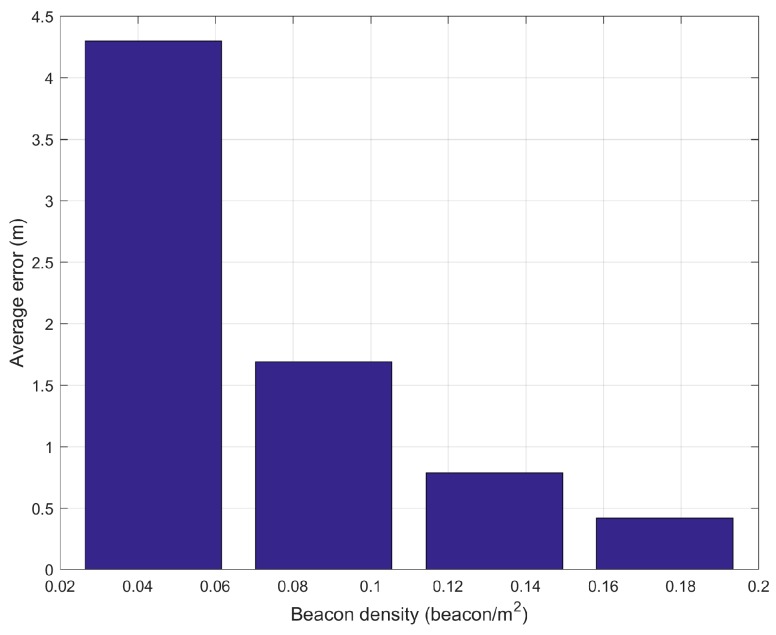
Average error vs. beacon density.

Following [68], we removed the beacons one by one and recalculated the average localization error to find the relationship between the localization error and beacon density. The results show that the average localization error is increasing as the density of beacons decreases (see Figure 20).

### 3.5. Evaluation

Our results are comparable or better than the results of other indoor positioning approaches that also use RSSI fingerprinting. For a realistic scenario of subjects walking in a poor wireless environment with many obstructions, Sung et al. [75] achieved the average localization error of 71.63 cm using a sigma-point Kalman particle filter (SKPF) with iBeacons. Chen et al. [76] achieved an accumulated error of under 1 m using the unscented Kalman filter algorithm with smartphone-integrated WiFi positioning. Lee et al. [77] achieved a recognition accuracy of 97.5% while using WiFi fingerprinting and Random Forest (RF) regression with an Arduino smart watch. Kanaris et al. [78] achieved an accurate localization error of 2.33 m while using an enhanced KNN positioning algorithm with a combination of WiFi and BLE systems. Chen et al. [79] achieved an average localization error of 1.11 m (for steady state only) while using a weighted fusion algorithm combined with a k-nearest neighbors (KNN) and unsupervised heuristic algorithm. Bi et al. [80] obtained the mean error of 2.2 m with an adaptive weighted KNN and twice affinity propagation clustering. 

The results of other authors in comparison with the current work are summarized in Table 1. Note that it is impossible to reproduce the real-life experimental conditions reported in other articles due to the very different test environments and equipment (sensing techniques) used in the respective works.

## 4. Concluding Remarks

The fingerprinting algorithm with fuzzy logic type-2 can be used as an indoor localization algorithm using Bluetooth Low Wnergy (BLE) beacons. The average error was 0.43 m (90% of measurements within 0.63 m), while an indoor navigation effectiveness of 98.2 ± 1% was achieved. The average error distance metrics of relative Hausdorff and relative Fréchet did not exceed 0.27%, while the maximal error did not exceed 0.45%, and the average navigation efficiency index (NEI) was higher than 86%. The fingerprinting localization requires pre-configuration, which is one of the main disadvantages of this method. Using a greater number of fingerprint points improves the result of the positioning. For larger rooms, more beacons are required for the correct operation of algorithms, while the maximum range of broadcasting in a real environment also should be taken into account. 

The presented approach is scalable and cost effective. The system consists only of relatively cheap Bluetooth Low Energy (BLE) beacons and a smartphone app to perform computations and issue spoken commands to the subjects carrying the smartphone. In terms of usability, we consider this solution as a good choice as evidenced by the acceptable values of the NEI score for the indoor navigation scenarios tested. The presented indoor navigation method could be applied for guidance to the visually impaired in a wide variety of real-world buildings such as office buildings, hospitals, hotels, airports, and museums.

Future work will include the exploration and improvement of fuzzy logic rules to increase the indoor localization accuracy even more.

## Figures and Tables

**Table 1:** Summary of related work in comparison with the method proposed in this paper. BLUE: Bluetooth Low Energy; GPS: Global Positioning System; MARG: magnetic, angular rate, and gravity; PDR: pedestrian dead reckoning; UWB: ultra wideband.

Method	Technology	Environment	Error in Meters	Error %
Segura et al. [17]	UWB	Room (6 m × 8 m)	0.2	N/A
Zhang et al. [19]	GPS, UWB, MARG	Business center	3.2	N/A
Sun et al. [21]	PDR	Atrium of Informatics Forum building (9.7 m × 5.94 m)	1.96	N/A
Yang et al. [24]	Stereo Camera	Room (8 m × 8.4 m × 4 m)	0.677	N/A
Qiu et al. [22]	Inertial/magnetic sensors, PDR	Room (approx. 20 m diameter, height 10 m), empty room	2.59	N/A
Meliones et al. [25]	Inertial dead-reckoning, BLE beacon	Floor (1640 m^2^)	N/A	2.53%
Liu et al. [16]	Peak intensities of lights	Supermarket (1000 m^2^)	N/A	0%
		Shopping mall (20 000 m^2^)	N/A	1.7%
		Office building (800 m^2^)	N/A	0%
Großwindhager et al. [18]	UWB	Office room (4 m × 6 m)	0.2	N/A
Zhou et al. [20]	Camera, PDR	Meeting room (16 m × 7.7 m)	0.56	N/A
Xu et al. [29]	grid-based, WiFi	Office (780 m^2^) Lab (1200 m^2^)	3.5m	N/A
Wang et al. [27]	WiFi, PDR	Floor in China University of Mining and Technology	4.99	N/A
Nguyen-Huu et al. [28]	PDR, WiFi fingerprint	Floor in Engineering building, Hallym University	2.40	N/A
Patel et al. [30]	BLE, Mapping/Poi	Office floor (1200 m^2^)	1.6	N/A
Proposed Method	BLE fingerprint, fuzzy logic	Floor (52.5m × 12.5 m)	0.43	N/A

## Appendix A

Fuzzy rules of Fuzzy logic type-1:

**IF** Euclidean Distance is Very Small **THEN** assign Very Large Weight

**IF** Euclidean Distance is Small **THEN** assign Large Weight

**IF** Euclidean Distance is Medium **THEN** assign Medium Weight

**IF** Euclidean Distance is Large **THEN** assign Small Weight

**IF** Euclidean Distance is Very Large **THEN** assign Very Small Weight

## Appendix B

Fuzzy rules for weight assignment, which include the Euclidean distance and signal strength:

**IF** Distance is Very Small **AND** RSSI is Close **THEN** assign Very Large Weight

**IF** Distance is Very Small **AND** RSSI is Near **THEN** assign Very Large Weight

**IF** Distance is Very Small **AND** RSSI is Far **THEN** assign Very Small Weight

**IF** Distance is Small **AND** RSSI is Close **THEN** assign Large Weight

**IF** Distance is Small **AND** RSSI is Near **THEN** assign Large Weight

**IF** Distance is Small **AND** RSSI is Far **THEN** assign Very Small Weight

**IF** Distance is Medium **AND** RSSI is Close **THEN** assign Small Weight

**IF** Distance is Medium **AND** RSSI is Near **THEN** assign Medium Weight

**IF** Distance is Medium **AND** RSSI is Far **THEN** assign Small Weight

**IF** Distance is Large **AND** RSSI is Close **THEN** assign Small Weight

**IF** Distance is Large **AND** RSSI is Near **THEN** assign Medium Weight

**IF** Distance is Large **AND** RSSI is Far **THEN** assign Large Weight

**IF** Distance is Very Large **AND** RSSI is Close **THEN** assign Very Small Weight

**IF** Distance is Very Large **AND** RSSI is Near **THEN** assign Small Weight

**IF** Distance is Very Large **AND** RSSI is Far **THEN** assign Very Large Weight

## Appendix C

**Table A1:** Linguistic variables for Euclidean distances.

Linguistic Variables	Membership Function Variable Values
Very Small Distance	0.0→0.0→2.5
Small Distance	0.0→2.5→5.0
Medium Distance	2.5→5.0→7.5
Large Distance	5.0→7.5→10.0
Very Large Distance	7.5→10.0→10.0

**Table A2:** Linguistic variables for weights.

Linguistic Variables	Membership Function Variable Values
Very Small Weight	0.0
Small Weight	2.5
Medium Weight	5.0
Large Weight	7.5
Very Large Weight	10.0

**Table A3:** Linguistic variables for distances.

Linguistic Variables	Membership Function Variable Values
Very Small Distance	0.0→0.0→2.5
Small Distance	0.0→2.5→5.0
Medium Distance	2.5→5.0→7.5
Large Distance	5.0→7.5→10.0
Very Large Distance	7.5→10.0→10.0

**Table A4:** Linguistic variables for RSSI.

Linguistic Variables	Membership Function Variable Values
RSSI-Close	−35.0→−28.0→−20.0
RSSI-Near	−66.0→−44.5→−31.0
RSSI-Far	−90.0→−75.0→−60.5

**Table A5:** Linguistic variables for output.

Linguistic Variables	Membership Function Variable Values
Very Small Weight	0.0
Small Weight	2.5
Medium Weight	5.0
Large Weight	7.5
Very Large Weight	10.0

**Table A6:** Fuzzy logic type-2 end rules.

Linguistic Variables	Upper Membership Function	Lower Membership Function
Very Small Distance	0.0→0.0→3.0	0.0→0.0→2.0
Small Distance	0.0→2.5→5.5	0.5→2.5→4.5
Medium Distance	2.0→5.0→8.0	3.0→5.0→7.0
Large Distance	4.5→7.5→10.0	5.5→7.5→9.5
Very Large Distance	7.0→10.0→10.0	8.0→10.0→10.0
